# Green Ultrasound-Assisted Synthesis of Surface-Decorated Nanoparticles of Fe_3_O_4_ with Au and Ag: Study of the Antifungal and Antibacterial Activity

**DOI:** 10.3390/jfb14060304

**Published:** 2023-06-01

**Authors:** Álvaro de Jesús Ruíz-Baltazar, Harald Norbert Böhnel, Daniel Larrañaga Ordaz, José Antonio Cervantes-Chávez, Néstor Méndez-Lozano, Simón Yobanny Reyes-López

**Affiliations:** 1 CONAHCYT-Centro de Física Aplicada y Tecnología Avanzada, Universidad Nacional Autónoma de México, Boulevard Juriquilla 3001, Santiago de Querétaro 76230, Mexico; 2 Centro de Geociencias, Universidad Nacional Autónoma de México, Boulevard Juriquilla 3001, Santiago de Querétaro 76230, Mexico; hboehnel@geociencias.unam.mx; 3 Minnesota Dental Research Center for Biomaterials and Biomechanical, School of Dentistry of Minnesota, Minneapolis, MN 55455, USA; daniel.larranaga@outlook.es; 4 Unidad de Microbiología Básica y Aplicada, Facultad de Ciencias Naturales, UAQ Campus Aeropuerto, Santiago de Querétaro 76140, Mexico; cervanteschavez@gmail.com; 5 Campus Querétaro, Universidad del Valle de México, Blvd. Juriquilla no. 1000 A. Del. Santa Rosa Jáuregui, Querétaro 76230, Mexico; nestor.mendez@uvmnet.edu; 6 Instituto de Ciencias Biomédicas, Departamento de Ciencias Químico-Biológicas, Universidad Autónoma de Ciudad Juárez, Anillo Envolvente del Pronaf y Estocolmo s/n, Zona Pronaf, Ciudad Juárez 32310, Mexico

**Keywords:** magnetic nanoparticles, magnetoplasmonic, antibacterial, antifungal, green synthesis, sonochemistry

## Abstract

This work proposes a sonochemical biosynthesis of magnetoplasmonic nanostructures of Fe_3_O_4_ decorated with Au and Ag. The magnetoplasmonic systems, such as Fe_3_O_4_ and Fe_3_O_4_-Ag, were characterized structurally and magnetically. The structural characterizations reveal the magnetite structures as the primary phase. Noble metals, such as Au and Ag, are present in the sample, resulting in a structure-decorated type. The magnetic measurements indicate the superparamagnetic behavior of the Fe_3_O_4_-Ag and Fe_3_O_4_-Au nanostructures. The characterizations were carried out by X-ray diffraction and scanning electron microscopy. Complementarily, antibacterial and antifungal assays were carried out to evaluate the potential properties and future applications in biomedicine.

## 1. Introduction

Nowadays, the research and development of magnetoplasmonic nanostructures have been widely studied due to their multiples applications in fields, such as catalysis, magneto-optics devices, energy storage, biosensors, drug delivery, photocatalysis, cancer treatment, hyperthermia, among others [1,2,3]. Commonly, magnetoplasmonic structures are constituted by ferromagnetic materials and noble metals, generally, gold and silver, as their remarkable properties are exhibited individually. In this sense, the catalytic, antibacterial, antifungal, biocompatible, and optoelectronic properties of noble metals, such as Ag and Au, allow them to be used in multiple fields, such as biomedicine, water treatment, catalysis, and photocatalysis, among others. Additionally, Au and Ag have been employed as electrochemical sensors, biosensors, and antibacterial and antifungal materials [4,5,6,7,8,9].

Concerning ferromagnetic materials, the superparamagnetic iron oxides nanoparticle’s (SPION’s) materials are the most commonly employed. The wide range of properties exhibited by the SPIONs allows their use in fields, such as medicine, optics, electronics, biology, and catalysis. However, the potentials applications of the magnetoplasmonic nanostructures depend directly on their morphology and size [4,10,11,12].

On the one hand, plasmonic materials, such as gold and silver, have been widely used due to their high biocompatibility and chemical and physical properties, which can promote mechanisms for the detection of components of biological interest. In addition, noble metals exhibit low toxicity, non-immunogenicity, and hydrophilicity and can be employed for selective drug delivery. Furthermore, the key optical features of the Au nanoparticles, such as surface plasmon resonance (SPR), SERS, and luminescence, allow gold nanoparticles to be used as biosensors and biomarkers. Specifically, the sensing mechanisms of the biosensors based on Au nanoparticles are supported by an optical phenomenon associated with the interaction between the conduction band of the Ag nanoparticles and the light photons, resulting in a conjunct oscillation of the ultraviolet–visible band and the valence electrons (SPR) [13,14,15]. In the case of the SERS methodology, it is based on an amplification of Raman scattering of the specific molecules absorbed on the metallic nanostructures or rough metal surfaces [16]. On the other hand, the magnetic materials, such as the SPION’s, the magnetic properties and functionality of these species have generated multiple applications in magnetic resonance imaging (MRI), specifically as magnetic contrast agents [17]. In this sense, new researchers are focused on developing nanostructures with iron oxide cores and some coatings or shells with hydrophilic properties with the finality to increase the magnetic susceptibility and consequently, their functionality [18]. Another important aspect is the kind of iron oxide employed in active targeting applications; the most common and functional iron oxides are magnetite (Fe_3_O_4_) and/or maghemite (γFe_2_O_3_) due to providing the particles with a magnetic resonance detectable signal, and the magnetic properties are similar to both species [19]. Likewise, the SPION’s size is directly related to its characteristic superparamagnetic behavior. It can be explained from the point of view of the aligned unpaired electrons spins (oriented magnetic domains), which depending on the structure and size of the SPION, can be classified as superparamagnetic if the material presents single domain crystals and exhibits a significantly larger magnetic susceptibility [20,21,22].

In this sense, the design and synthesis of magnetoplasmonic nanostructures acquire a preponderant place in nanotechnology. For this reason, the development of synthesis routes that lead obtains homogeneous structures in composition, morphology, and size and nowadays is an interesting topic [23,24,25]. In this regard, the implementation and search for ecological alternatives for the synthesis of nanostructures has been focused on green chemistry due to these methodologies to eliminate or significantly reduce the use and generation of toxic chemical reagents and hazardous substances [26,27,28,29]. The green synthesis of nanostructures is commonly based on the use of extracts from plants, bacteria, algae, fungi, roots, and yeasts, among others, as reducing agents during green synthesis [4,30,31]. This is due to the nanostructures containing high levels of antioxidants, phenolic compounds, flavonoids, isoflavones flavanones, flavones, flavanols, and anthocyanidins [17,18,19]. Additionally, the implementation of ultrasound-assisted green synthesis represents a great alternative for obtaining bifunctional system nanostructures composed of SPIONs, Au, and Ag [19,25,32].

In concrete form, the sonochemical synthesis of magneto plasmonic nanoparticles promotes the dispersion of the reactive and reactants during the synthesis process. Many reports indicate that it is possible to obtain bifunctional systems, such as those composed by SPIONs, Au, and Ag [22,33,34], through sonochemical synthesis.

On the other hand, the biomedical applications of magnetoplasmonic materials have been accentuated, specifically in the antibacterial and antifungal fields, due to the actual threats in health topics due to bacterial multidrug resistance. This is derivative to the excessive and imprudent use of antibiotics complicating the control of the epidemic [18,35]. In this sense, the increasing rate of microbial resistance has propelled and motived the search for and development of efficient therapies. In this regard, the incorporation of phytochemicals contained in plants, also known as organic antibiotics, have exhibited significant antibacterial properties. According to their organic compounds, the organic antibiotics can be classified as alkaloids, terpenoids, polyphenols, and sulfur-containing compounds [35,36]. These compounds are commonly found on plants, such as *Berberis vulgaris*, *Piper nigrum*, *Arctium lappa*, *Carum carvi*, *Rhamnus purshiana*, *Matricaria chamomilla*, *Eucalyptus globulus*, among others. Specifically, components derived from the alkaloids, such as reserpine, piperine, berberine, chanoclavine, conessine, solasodine, evocarpine, and tomatidine, hves shown exceptional results against *Staphylococcus aureus*, *Streptococcus agalactiae*, *Micrococcus luteus*, and *Escherichia coli*, among others. Regarding the terpene compounds, for instance, farnesol, nerolidol, and dehydroabietic acid, it has been observed that these compounds manage to generate a cell membrane disturbance against *S. aureus* bacteria [18,35]. In addition, phenolic compounds, such as resveratrol, baicalein, biochanin a and formononetin, have shown reductions in the minimum inhibitory concentration values of antibacterial agent against mycobacterium smegmatis and campylobacter jejuni, among others. Other important eukaryotic pathogens, such as the fungi, also show a threat due to their intrinsic characteristics of adaptations in metabolism and morphogenesis that enable fungi to generate problems in human health. It has been reported that eukaryotic microorganisms, such as phylum Ascomycota specie, contain virulent fungi, such as coccidioides, coccidioides, clastomyces, histoplasma, scedosporium, fusarium, aspergillus, and candida species [4,37].

The latter species is a priority study objective since most human infections caused by fungal pathogens are attributed to this species, specifically, *Candida albicans*, *Candida glabrata*, which exhibits significant drug resistance, and *Candida auris*, which has recently been considered the new threat to global public health. Furthermore, emerging species, such as *Candida krusei*, *Candida tropicalis*, and *Candida parapsilosis*, have also generated particular interest [37].

Based on the above arguments, it is necessary to develop alternatives to address this problem. Nanoparticles (NPs) of noble metals and metal oxides have proven to be functional alternatives to address antibiotic and antifungal problems. In this regard, the use of this systems in antibacterial and antifungal applications has been widely reported recently due to actually slowing the approved rate of antibiotics and antifungals [4,38,39,40,41]. Systems composed of iron oxides, Au, and Ag nanoparticles have been probed again microorganism, such as *E. coli*, *S. aureus*, *Candida parapsilosis*, *C. glabrata*, and *C. albicans* [3,5,42]. Because these types of nanoparticles can penetrate the cell membranes in bacteria and fungi, altering cell function, on the other hand, the high surface energy of the particles promotes the antibacterial and antifungal effect [4,43,44,45].

This work intends to focus on the problem of the resistance of bacteria and fungi to traditional antibiotics, generating novel alternatives from the point of view of the synthesis of the proposed materials, which are obtained through the use of green chemistry. Emphasizing the innovation of the green methodology, which offers an alternative considerably more economical and ecological, since the use of organic extracts, such as *Piper auritum,* during the synthesis methodology of magnetoplasmonic nanostructures and the use of ultrasound as a complementary tool allows us to propose a hybrid and functional methodology for obtaining this type of nanostructures.

The use of magnetoplasmonic materials are novel due to their broad and synergistic properties (plasmonic and magnetic). The structural and chemical characterization confirms the synthesis of the Fe_3_O_4_, Fe_3_O_4_-Au, and Fe_3_O_4_–Ag structures. The antibacterial and antifungal activities of the obtained nanostructure were evaluated against Gram-positive bacteria, *Staphylococcus aureus* (ATCC 6538), and *Escherichia coli* (ATCC 8739) as Gram-negative. For eukaryotic, yeast cells of *Candida parapsilosis*, *C. glabrata*, and *C. albicans* (ATCC 10231) were selected. Significant results were found regarding the antibacterial and antifungal activities of the Fe_3_O_4_-Au and Fe_3_O_4_-Ag samples. The synergistic effect of noble metals with iron oxides as an antifungal and antibacterial alternative is presented and discussed in this work.

## 2. Materials and Methods

### 2.1. Green Synthesis of Magneto-Plasmonic Systems

The synthesis of the magnetoplasmonic systems (Fe_3_O_4_, Fe_3_O_4_-Au, and Au-Ag) were carried out from *Piper auritum* extract and assisted by ultrasound. In the case of the Fe_3_O_4_ nanoparticles, a FeCl3.6H_2_O/FeCl2.4H2O solution in a ratio of Fe (III)/Fe (II):2 was employed as the precursor agent. The reducing agent from the Piper auritum extract were obtained with a mixture of 200 mL of deionized water with 15 g of Piper auritum leaves. The mixture obtained was heated at 100 °C for 10 min.

The synthesis of Fe_3_O_4_-Au nanoparticles was carried out in an analogous form to the methodology reported in a previous work [24]. Briefly, the reducing agent from the *Piper auritum* extract was obtained with a mixture of 200 mL of deionized water with 15 g of *Piper auritum* leaves. The mixture obtained was heated at 100 °C for 10 min. On the other hand, the metallic precursor was prepared with a HAuCl_4_·3H_2_O solution at 50 mM. Posteriorly, the precursor and the reducing agent were mixed and ultrasonicated at 42 kHz for 15 min. Finally, a FeCl_3_.6H_2_O/FeCl_2_.4H_2_O solution in a ratio of Fe (III)/Fe (II):2 was added to the previous mixture and assisted with ultrasonic agitation for 60 min. (Digital Ultrasonic Cleaner, Brand: Kendall, Model: CD-4820, Pontiac, MI 48341, USA; 42 kHz of frequency and 160 W). The pH of the synthesis reaction was adjusted to 11 by adding a NaOH solution. The Fe_3_O_4_-Au nanoparticles obtained were washed in an isopropyl alcohol solution and centrifuged. The colloidal solution of the Fe_3_O_4_-Au nanoparticles was dried for the subsequent procedures of the chemical–structural characterization and properties evaluations. On the other hand, the Fe_3_O_4_-Ag nanoparticles were obtained in similar form of Fe_3_O_4_-Au nanoparticles. In this case, a solution of AgNO_3_ at 50 mM was employed as a precursor. This solution was mixed and sonicated with the reducing agent (*Piper auritum* extract). Posteriorly, a previously prepared FeCl_3_.6H_2_O/FeCl_2_.4H_2_O solution was added to the precursor/reducing agent solution. In the final stage of the process, the mix obtained was heated at 40 °C under ultrasonic agitation for 60 min. The solution obtained was washed with isopropyl alcohol and centrifuged at 4000 rpm for a period of 15 min. (Thermo Scientific brand, Waltham, MA, USA; Heraeus X1R model) at 4 °C. In the final stage, the solution was decanted, and the solids obtained were lyophilized.

### 2.2. X-ray Diffraction Analysis

The structural characterization of the Fe_3_O_4_, Fe_3_O_4_-Au and Au-Ag samples were carried out in a Rigaku Ultima IV X-ray diffractometer (Suite 475 Austin, TX 78717, USA). In a range of exploration of 10–80 degrees (2θ) with Cu-Kα radiation (λ = 1.54° A). The step of the analysis was 5° per minute and sampling every 0.02.

### 2.3. Scanning Electron Microscopy (SEM)

The morphology and the configuration of the Fe_3_O_4_, Fe_3_O_4_-Au, and Au-Ag samples were determined by high-resolution scanning electron microscopy (HRSEM) through a Hitachi SU-8230 Scanning Electron Microscope (2535 Augustine Drive, Santa Clara, CA 95054, USA). The microscope was equipped with a cold field emission device.

### 2.4. Chemical Characterization

The chemical composition of the samples was determined by energy-dispersive X-ray spectroscopy (EDS). The elemental compositions of the samples were collected with a Bruker X Flash 6/60 system coupled to the Hitachi SU-8230 Scanning Electron Microscope.

### 2.5. Antibiotic Assay

In order to determine if the magnetite nanoparticles showed antimicrobial activity, we tested its effect by means of the Kirby Bauer method using procaryotic and eukaryotic cells. Briefly, 5 mg of nanoparticles S7, S14, and S15 were weighed independently, and 1 mL of distilled water was added to each one. As a Gram-positive representative bacteria we chose *Staphylococcus aureus* (ATCC 6538) and *Escherichia coli* (ATCC 8739) as a Gram-negative representative bacteria. For eukaryotic, yeast cells of *Candida parapsilosis*, *C. glabrata* (kindly donated by Elva T. Aréchiga Carvajal, Universidad Autónoma de Nuevo León) and *C. albicans* (ATCC 10231) were selected.

Bacteria strains were grown in nutrient broth (NB) media and were incubated at 37 °C for 20 h. For yeast, YPD (2% yeast extract, 1% peptone 2% dextrose) media was used and were incubated at 28 °C for 24 h. Optical density (OD) was scored at 595 nm (Thermo Scientific Genesys 105-UV-Vis). Then, OD of the cultures was adjusted to 1 OD unit using fresh NB or YPD media. Next, a volume of 200 μL was spread with the aid of sterile glass beads on Muller Hinton agar (bacteria) or YPD (yeast). Later on, filter disks of Whatman paper were placed on the plates, and 100 μL of each nanoparticle were dispensed. As the control, a volume of 100 μL of sterile distilled water was dispensed on a filter paper disk. Plates were dry in the hood, then incubated at 37 °C (bacteria) or 28 °C (yeast) for 24 h. Next, the inhibition halo sizes were measured, and plates were photographed. Experiments were conducted by triplicate. Data were analyzed by one way ANOVA.

## 3. Results and Discussion

### 3.1. Materials Characterization

#### Scanning Electron Microscopy

**Figure 1:**
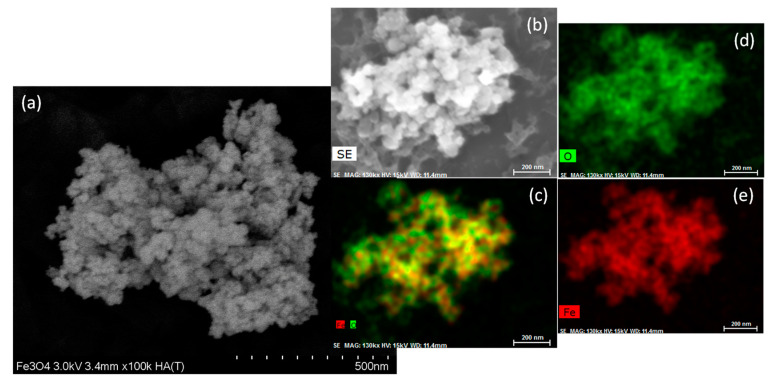
Scanning electron microscopy SEM images of the Fe_3_O_4_ nanoparticles: (**a**) operated in transmission mode at 3.0 kV, (**b**) secondary electrons image, (**c**) energy dispersive spectrometry (EDS) general mapping(Image sizes are annotated by the scale bar in the lower right corner of the respective image), and (**d**,**e**) elemental mapping of oxygen and iron, respectively.

The scanning electron microscopy SEM (HA-T) of the Fe_3_O_4_ is shown in Figure 1a, which indicates the semispherical morphology and a high agglomeration degree. This fact can be attributed to the strong interaction generated on the Fe_3_O_4_ nanoparticles surface. On the other hand, the distribution particle size observed was 35 nm approximately. The subsequent X-ray diffraction analysis complements the crystallite size of the samples. Figure 1b shows in detail a SE image of the Fe_3_O_4_ sample. In this image, the semispherical morphology, the particle distribution size, and the agglomeration of the Fe_3_O_4_ NPs are corroborated. The Energy dispersive spectroscopy (EDS) mapping of the Fe_3_O_4_ is presented in Figure 1c. The constitutive elements (Fe and O) of the Fe_3_O_4_ NPs were fully identified and marked. In complementary form, Figure 1d,e, illustrate the elemental mapping of the oxygen an iron, respectively.

**Figure 2:**
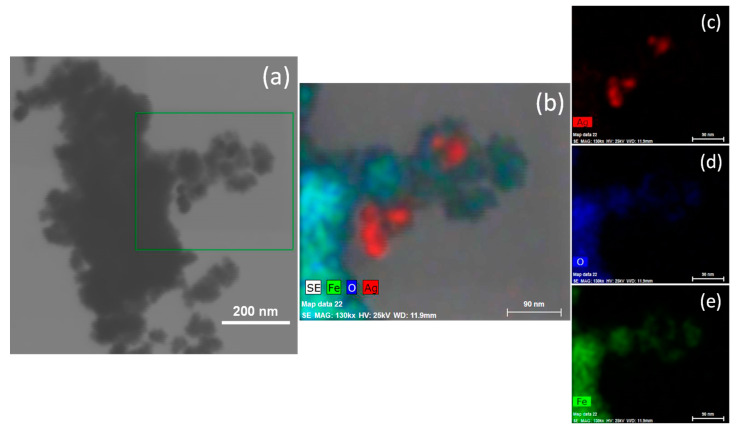
(**a**) SEM micrographs of the Fe_3_O_4_-Ag plasmonic nanoparticles: (**b**) elemental mapping of the Fe_3_O_4_-Ag nanostructures obtained, and (**c**–**e**) individual mappings of the Ag, O, and Fe elements, respectively (Image sizes are annotated by the scale bar in the lower right corner of the respective image).

In regard to the Fe_3_O_4_-Ag NPs, Figure 2a shows an SEM image of the sample, where similar characteristics to the Fe_3_O_4_ sample were observed in terms of morphology, size distribution, and agglomeration degree. However, the silver added to the Fe_3_O_4_ synthesis showed a partial nucleation on the Fe_3_O_4_ NPs. Fe_3_O_4_-Ag NPs are given as the decorated type. The Ag NPs nucleated on the magnetite surface exhibits an approximated size of 25 nm. Figure 2b shows the EDS mapping of the sample Fe_3_O_4_-Ag, which corroborates the Ag presence. The individual mapping element associated with Ag, O, and Fe are presented in Figure 2c–e, respectively.

**Figure 3:**
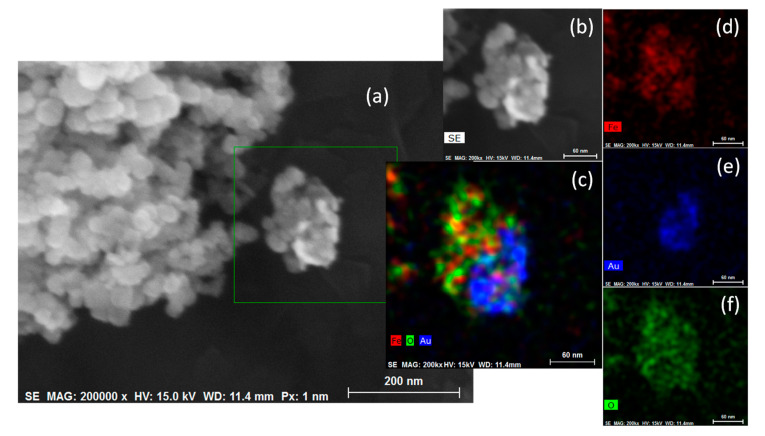
(**a**,**b**) Secondary electron micrographs of the Fe_3_O_4_-Au nanoparticles: (**c**) EDS mapping of Fe_3_O_4_-Au sample obtained at 15 kV and (**d**–**f**) elemental mappings of Fe, Au, and O (Image sizes are annotated by the scale bar in the lower right corner of the respective image).

Finally, the structure and morphology of the Fe_3_O_4_-Au samples are described in Figure 3a–f. Specifically, Figure 3a shows an SEM image of the Fe_3_O_4_-Au sample, which describe in general form, the distribution and agglomeration degree of the Fe_3_O_4_-Au NPs. In this figure it is possible to appreciate the Fe_3_O_4_ and Au configuration as a decorated type. Figure 3b illustrate in detail an individual particle of Fe_3_O_4_-Au. The specific localizations of the Fe, O, and Au are appreciated (Figure 3c). As part of the chemical characterization, elemental EDs mapping of Fe, Au, and O are showed in Figure 3d–f, respectively.

In this sense, and according to the synthesis method, the Fe_3_O_4_ act as nucleation sites, which promote the Au NPs growth. For this reason, particles decorated type were obtained.

### 3.2. X-Ray Diffraction Analysis and Williamson-Hall Methods (W-H)

**Figure 4:**
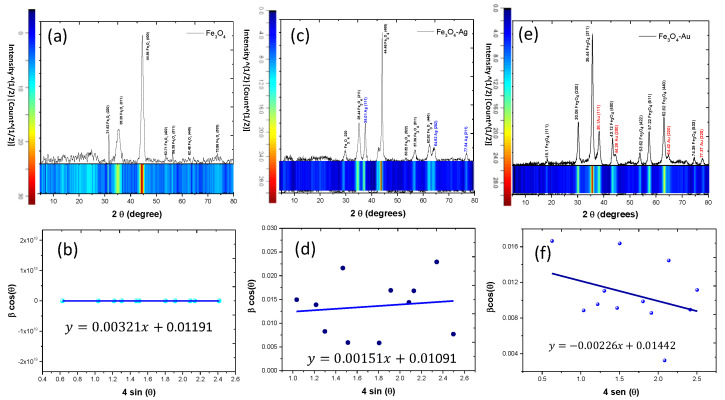
X-ray diffraction patterns and Williamson–Hall plots corresponding to the (**a**,**b**) Fe_3_O_4_, (**c**,**d**) Fe_3_O_4_-Ag, and (**e,f**) Fe_3_O_4_-Au nanoparticles.

In order to elucidate the structure of the Fe_3_O_4_, Fe_3_O_4_-Ag, and Fe_3_O_4_-Au samples, XRD analysis was carried out. XRD patterns and their respective Williamson–Hall plots are described in Figure 4a–f. In general form, the Williamson–Hall methods can be employed to calculate the crystallite size from the XRD patterns. In this work the uniform deformation model is considered to calculate the crystallite size and strain in the Fe_3_O_4_, Fe_3_O_4_-Ag, and Fe_3_O_4_-Au nanoparticles.

#### Uniform Deformation Model (UDM)

In this method, the central idea is the fact that the XRDs are not only influenced by the crystal size but also by the strains and deformations of the crystal lattice of the material. Intrinsically, the W-H analysis is a simplified integral breadth method through which it is possible to discern between the peaks induced by deformation and the peaks associated with the crystallite sizes. In this sense, the individual contributions observed on the line broadening in the diffraction angle (2θ) can be expressed as [46,47]:

(1) $$\beta_{hkl}=\beta_{s}+\beta_{D}$$

where *β_hkl_* is the full width at half maximum (FWHM) of the observed peaks in the X-ray diffraction patterns. Consequently, *βs* and *β_D_* are the contributions to the width peak attributed to the size and strain, respectively. It is assumed that the strain is uniform throughout a specific crystallographic direction. The mathematical expression for the uniform deformation model (UDM) is defined as [47,48]:

(2) $$\beta_{hkl}=\frac{k\lambda }{D\cos\theta }+4\varepsilon \tan\theta$$

Rewriting Equation (3):

(3) $$\beta_{hkl}\cos\theta =\frac{k\lambda }{D}+4\varepsilon \sin\theta$$

Based on Equation (4), the values of *β* cos *θ* and 4 sin *θ* can be graphed from the linear fit and the respective slope and intercept can be used to calculate the crystallite size and strain of the samples, respectively. Figure 2b,d,f show the (UDM) of the Fe_3_O_4_, Fe_3_O_4_-Ag, and Fe_3_O_4_-Au, respectively.

From the obtained values of the slope and intercept in the Williamson–Hall plots, the crystallite sizes calculated are 12.93, 14.12, and 10.58 nm for the Fe_3_O_4_, Fe_3_O_4_-Ag, and Fe_3_O_4_-Au NPs, respectively. These results are consistent with the SEM images. Nevertheless, the plasmonic systems show a high agglomeration degree; nonetheless, in the SEM image it is possible to observe individual particulates with crystallite sizes approximate to the calculated values by the Williamson–Hall methods. Therefore, it is possible to affirm that the theoretical calculations associated with the crystallite sizes can describe and confirm the sizes distribution of the nanoparticles observed by SEM. With respect to the synthesis methodology, it is possible to affirms that the Fe_3_O_4_, Fe_3_O_4_-Au, and Fe_3_O_4_-Ag structures are formed by the ultrasound-assisted methodology and the use of the Piper auritum extract as the reducing agent. The nucleation rates of the iron, gold, and silver, as well as the reaction time are consistent with the Fe_3_O_4_, Fe_3_O_4_-Au and Fe_3_O_4_-Ag obtention. The configuration surface-decorated nanoparticles are fully identified and are confirmed chemical and structurally. The following magnetic measures presented can confirm the magnetic behavior as a function of the SPION’s type obtained.

### 3.3. Magnetic Properties

**Figure 5:**
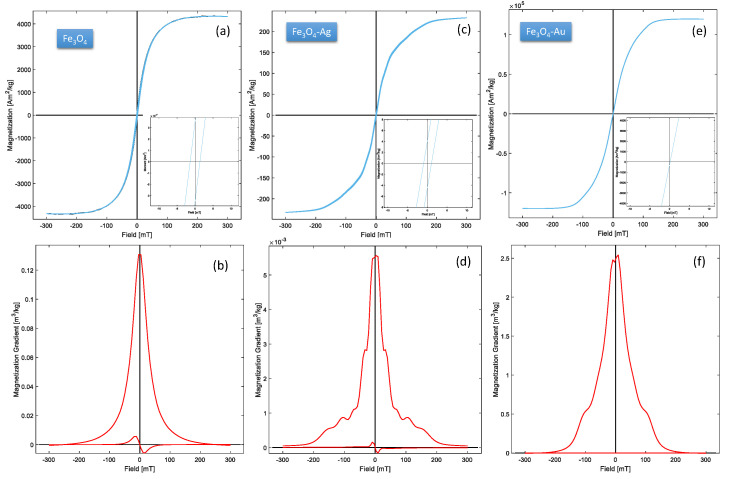
(**a**,**c**,**e**) Hysteresis loops of the Fe_3_O_4_, Fe_3_O_4_-Ag, and Fe_3_O_4_-Au NPs. (**b**,**d**,**f**) Magnetization gradients plots of the magnetoplasmonic systems of Fe_3_O_4_, Fe_3_O_4_-Ag, and Fe_3_O_4_-Au, respectively.

In order to establish the magnetic behavior of the Fe_3_O_4_, Fe_3_O_4_-Ag, and Fe_3_O_4_-Au NPs, hysteresis loop of the materials were carried out. Figure 5a–f show graphically the magnetic properties observed for each sample. Specifically, Figure 5a,c,e describe the hysteresis loop from the Fe_3_O_4_, Fe_3_O_4_-Ag, and Fe_3_O_4_-Au nanoparticles, respectively. Table 1 shows the values of the magnetics parameters, such as saturation magnetization (Ms), remanent magnetization (Mrs), and coercive field associated with the Fe_3_O_4_, Fe_3_O_4_-Ag and Fe_3_O_4_-Au samples.

In this sense, it is important to note that the Bc value is nearly zero in three cases; consequently, a superparamagnetic behavior can be considered. The incorporation of the noble metals (Ag and Au) to the iron oxide, in this case, promotes a clear tendency to the superparamagnetic behavior of the material. Thus, the observed diminution in the Bc value as a composition function of the samples can be attributed in the first instance to the crystallite size and to the ordering of the nanodomains (magnetic anisotropy) [20,25,49,50]. That is to say, the interactions between the canted spins at the iron oxide surface associated with the crystallite size can determine the superparamagnetic behavior of the Fe_3_O_4_, Fe_3_O_4_-Ag, and Fe_3_O_4_-Au samples. Finally, Figure 5b,d,f, show the magnetization gradients, Mr and Mrs, corresponding to magnetoplasmonic Fe_3_O_4_, Fe_3_O_4_-Ag, and Fe_3_O_4_-Au NPs. These graphs illustrate and support the parameters obtained from the hysteresis loop, where the approximate superparamagnetic behavior is confirmed. On the other hand, the Ms value associated with the Fe_3_O_4_-Au sample exhibits a considerable increase; this fact can be related to factors, such as magnetic domains, particle sizes, the quantity of Au incorporated to the decorated, and mainly to the synergic effect of the Fe_3_O_4_ and the Au since the Fe_3_O_4_-Au NPs are composed of two species, generating regions aligned in the spins on the surface and interface of the particles [33].

### 3.4. Antibacterial and Antifungal Activity Assay

**Figure 6:**
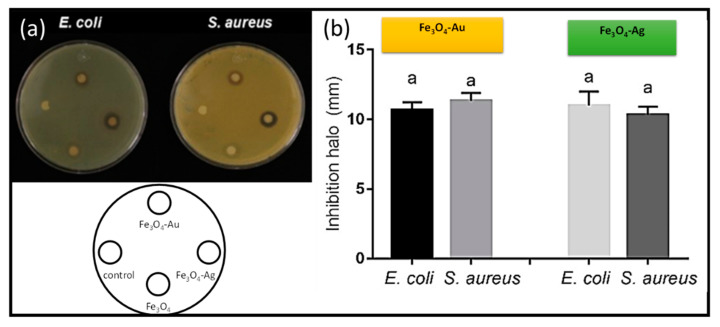
(**a**) Inhibition zone test obtained from the Fe3O4-Ag, and Fe3O4-Au nanoparticles against *S. aureus* and *E. coli* (sample distribution in the antibacterial assays is inserted at the bottom of the image) and (**b**) antibacterial activity graphs of the Fe_3_O_4_, Fe_3_O_4_-Ag, and Fe_3_O_4_-Au nanoparticles against the Gram-positive *S. aureus* and Gram-negative *E. coli* bacteria. (The letters a on the graphs describe the statistical differences between the data).

The Fe_3_O_4_-Ag and Fe_3_O_4_-Au nanoparticles showed antibacterial activity against the Gram-positive *S. aureus* and Gram-negative *E. coli* bacteria. *S. aureus* produces infections, such as bacteremia, endocarditis, pneumonia, and sepsis. Similar inhibitory halos were obtained with both kinds of nanoparticles (Figure 6), suggesting a general way of action of this material, considering the cell wall differences presented between Gram-positive and -negative bacteria [13,51].

**Figure 7:**
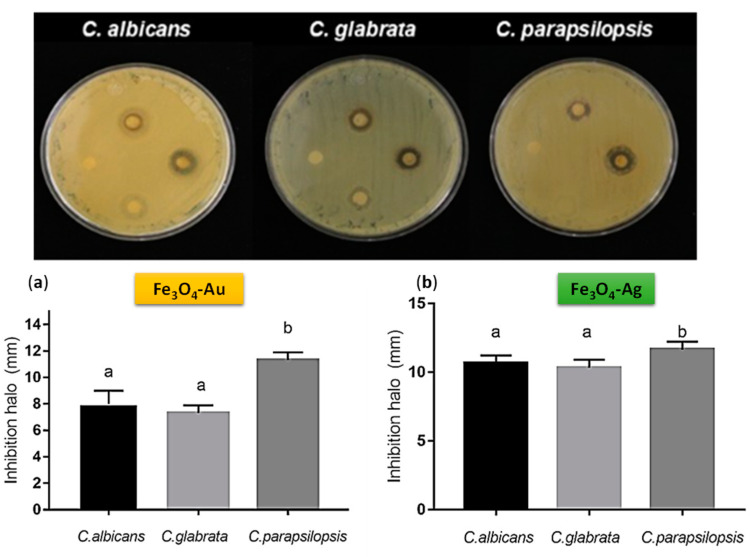
Antifungal activity against *C. albicans*, *C. glabrata*, and *C. parapsilopsis* of the (**a**) Fe_3_O_4_-Au and (**b**) Fe_3_O_4_-Ag samples obtained by green synthesis route (The letters a and b on the graphs describe the statistical differences between the data).

On the other hand, regarding the fungicide effect of Fe_3_O_4_ particles in eukaryotic cells, it is important to mention the fact that three Candida species were tested: *C. albicans*, *C. parapsilosis*, and *C. glabrata*, all human-life-threatening pathogens due to the high morbidity and mortality [52,53]. In our experiments, the three species were similarly affected either by Fe_3_O_4_-Ag or Fe_3_O_4_-Au nanoparticles (Figure 7). Since the cell wall is the first structure to establish contact with the environment and with the host, it is important to stress the importance of the data obtained with Candida regarding that the cell wall is similar in composition (glucans, mannans, or chitin), but the architecture is different, changing in this way the interaction with the host [54,55].

Nevertheless, prokaryotic or eukaryotic cells were not affected by the presence of magnetite nanoparticles since no growth inhibition was observed (data not shown), suggesting that the antibiotic effect observed was due to the action of metallic ions or to the interaction between magnetite and the metal. Our results highlight the possibility of using Fe_3_O_4_-Ag or Fe_3_O_4_-Au nanoparticles as an alternative approach to control the growth of Candida strains either sensitive or resistant to antibiotics, as well as *S. aureus*. Likewise, these types of nanostructures could be used in dental implants whenever biocompatibility tests are carried out. The high antibacterial and antifungal activity observed by these systems (Fe_3_O_4_-Au and Fe_3_O_4_-Ag) offer the possibility to be employed as nanocarriers of drugs in dental applications [56,57,58,59].

## 4. Conclusions

The magnetoplasmonic systems Fe_3_O_4_- (Ag and Au) synthesized by green route proposed in this work exhibits significant magnetic and antibiotic properties. On the one hand, the nanoparticles obtained by green route structurally show a decorated type in the case of the systems Fe_3_O_4_-Ag and Fe_3_O_4_-Au. The structures of the samples were fully identified and associated with Fe_3_O_4_, Fe_3_O_4_-Ag and Fe_3_O_4_-Au. On the other hand, regarding to the magnetic properties of the nanoparticles obtained, a near superparamagnetic behavior was observed in Fe_3_O_4_, Fe_3_O_4_-Ag, and Fe_3_O_4_-Au. Finally, the antibiotics assay shows potential applications of the Fe_3_O_4_-Ag and Fe_3_O_4_-Au as antibacterial and antifungal materials. Specifically, our results highlight the possibility to use Fe_3_O_4_-Ag or Fe_3_O_4_-Au nanoparticles as alternative approaches to control the growth of Candida strains either sensitive or resistant to antibiotics, as well as *S. aureus*.

This is presumably due to the presence of the metallic nanoparticles and the synergic effect of the magnetite and the noble metals and their respective interactions with the prokaryotic or eukaryotic cells.

## Figures and Tables

**Table 1:** Magnetic parameters measured from the Fe_3_O_4_, Fe_3_O_4_-Ag, and Fe_3_O_4_-Au nanoparticles.

**Sample**	**Ms** **(Am^2^/kg)**	**Mrs** **(Am^2^/kg)**	**Bc** **(mT)**
Fe_3_O_4_	4340	147	1.4
Fe_3_O_4_-Ag	217	4.25	1.0
Fe_3_O_4_-Au	120,000	304	0.2

## Data Availability

No new data were created or analyzed in this study. Data sharing is not applicable to this article.
